# Datasets for corporate social responsibility index of Jordanian industrial and service sector firms

**DOI:** 10.1016/j.dib.2021.107574

**Published:** 2021-11-13

**Authors:** Moawiah Awad Alghizzawi, Mayada A. Youssef, Mohammed Abu Zraiq

**Affiliations:** a Department of Accounting and Finance, United Arab Emirates University, Abu Dhabi, Al-Ain, United Arab Emirates; b Faculty of Economics and Muamalat, Universiti Sains Islam Malaysia, Nilai, Negeri Sembilan 71800, Malaysia

**Keywords:** Corporate social responsibility, Amman stock exchange, Industrial sector, Service sector, Jordan

## Abstract

Detailed data concerning firm-level corporate social responsibility initiatives enforced by the Jordan Securities Commission (JSC) were covered in the present study. Panel data from 100 Jordanian firms listed on the Amman Stock Exchange (ASE) in the service and industrial sectors were utilised in this research. The study was undertaken between 2017 and 2018. Beside, data retrieved from the firms' annual reports are presented in this study. The official website of ASE and the websites of each firm involved were sources for the downloaded annual reports. The data was used efficiently by the researchers to establish and compute a corporate social responsibility index involving internal attributes. The corporate social responsibility index comprised three unique attributes, notably "Philanthropy, Community and Environment". Thus, the unweighted corporate social responsibility has an essential feature of "easy for replicable and modifiable" that enabled the researchers to evaluate firms according to an aggregate index score.

**Specifications Table**

**Table:** 

Subject	Accounting
Specific subject area	Corporate Social Responsibility
Type of data	Table (Excel file)
How data were acquired	The dataset was manually collected from the annual reports of firms by using content analysis, annual reports downloaded from the official website of ASE or from the official website from each firms.
Data format	Raw, Filtered
Parameters for data collection	Industrial and service sector firms listed in ASE and had available information for the current study during the entire period study. These firms sharing the same requirements as imposed by the JSC. This study excluded the financial firms' sector due to variations in the regulatory framework and differ in structure, methods and accounting practices from the sample.
Description of data collection	Secondary Data: The data were hand-collected from the annual reports of each firm for the pertaining years that were published at official website of ASE or from the official website from each firm. Depending on content analysis of 100 Industrial and service sector firms two years observations (2017-2018).
Data source location	United Arab Emirates University (UAEU). Firms’ annual reports obtained from ASE (http://www.ase.com.jo), Securities Depository Center (https://www.sdc.com.jo/english/) and firms’ official websites.
Data with this article.	The data were attached within this article

**Value of the Data**

• Considering the lack of a systematic procedure or standardised system to assess firm adherence with the nation's corporate governance code pertaining to corporate social responsibility, the dataset comprises distinct characteristics and extensive governance practices in Jordan's industrial and service sectors.
• The data could be beneficial to investors, researchers and businesses in determining whether a company complies with corporate governance standards related to corporate social responsibility.
• The data could be used to easily develop a corporate social responsibility index to assess and rank non-financial companies' adherence to corporate social responsibility laws. Investors can use this rating to contrasts between firms and make sound investment choices.
• Researchers can use the data to study the correlation between corporate social responsibility indices and businesses' operational and financial success.

## 1 Data Description

The cross-sectional data encompassed observations of 100 industrial and service firms available in the ASE database for two years between 2017 and 2018. Furthermore, additional data were obtained from the Securities Depository Center (SDC). The codes in the excel file are representing the names of the selected firms in ASE. The data were employed to create a corporate social responsibility index according to a binary scale of attributes (Refer to Table 2). Beside that, the data involved Return on Assets (ROA) and Return on Equity (ROE) in indicating to firms’ performance (Refer to Table 5). The study involved 98 firm-year observations from the industrial sector, which were divided into nine segments. Beside, another 102 firm-year observations were from the services sector and segregated into eight segments. Table 1 displays the data description comprehensively.

**Table 1:** Descriptive statistics for the frequency of corporate social responsibility attributes in service and industrial sectors.

	Industrial sector firms	Obs.	Freq.	%
1	Pharmaceutical and Medical Industries	8	3207	8.17
2	Chemical Industries	16	2321	16.02
3	Food and Beverages	20	1120	20.555
4	Tobacco and Cigarettes	4	891	4.165
5	Mining and Extraction Industries	24	1513	24.64
6	Engineering and Construction	12	1266	11.856
7	Electrical Industries	6	1062	6.29
8	Textiles, Leathers and Clothing's	6	1939	5.894
9	Printing & Packaging	2	1373	2.41

	**Total**	98	14692	100%

	**Services sector firms**			
1	Health Care Services	9	1304	8.23
2	Educational Services	13	1716	12.87
3	Hotels and Tourism	18	2041	17.75
4	Transportation	18	2314	17.75
5	Technology and Communication	6	1304	5.94
6	Media	4	1047	3.92
7	Utilities and Energy	13	1692	12.85
8	Commercial Services	21	2491	20.69

	Total	102	13910	100%
	Total for all segments	200	28602	

The frequency of corporate social responsibility aspects in Jordanian firms from the non-financial sector is exhibited in Table 1. The frequency has been segregated according to major segments that had been categorised to services firms (e.g., Media, Utilities & Energy, Educational Services, Commercial Services, Hotels & Tourism, Health Care Services, and Transportation, Technology & Communication) and industrial sector firms (e.g., Electrical Industries, Chemical Industries, Food & Beverages, Engineering & Construction, Pharmaceutical & Medical Industries, Mining & Extraction Industries, Printing & Packaging, Textiles, Leathers & Clothing's and Tobacco & Cigarettes).

## 2 Experimental Design, Materials and Methods

The researchers gathered the three corporate social responsibility elements for each individual industrial and service firm. The governance elements presented in Tables 2–4 were transformed to a binary variant. If a firm had the attribute, it is entered as "1," whereas it is encoded as "0" if the attribute was not present. The corporate social responsibility index for industrial and service Jordanian firms was build based on dichotomous variables. As per Alshannag et al. [1], every index property is established on a binary scale with a value of 1 or 0. The value of 1 denotes compliance to the attribute, and 0 indicates non-compliance.

**Table 2:** Descriptive Statistics analysis elements used to construct the corporate social (Philanthropy) responsibility Index in Jordanian industrial and service firms.

A.	Disclosure and Transparency (D & T)	Freq.	Mean
1.	Firms do not have a Philanthropy disclosure related information in the annual report	113	43%
2.	Firms have a Philanthropy disclosure related information in the annual report	87	57%

**Table 3:** Descriptive Statistics analysis elements used to construct the corporate social (Environment) responsibility Index in Jordanian industrial and service firms.

A.	Disclosure and Transparency (D & T)	Freq.	Mean
1.	Firms do not have an Environment disclosure related information in the annual report	142	29%
2.	Firms have an Environment disclosure related information in the annual report	58	71%

**Table 4:** Descriptive Statistics analysis elements used to construct the corporate social (Community) responsibility Index in Jordanian industrial and service firms.

A.	Disclosure and Transparency (D & T)	Freq.	Mean
1.	Firms do not have a Community disclosure related information in the annual report	113	57%
2.	Firms have a Community disclosure related information in the annual report	87	43%

These elements used to construct the Corporate Social Responsibility Index for 100 Jordanian industrial and service firms during the period (2017-2018).

**Table 5:** Descriptive Statistics analysis elements used to construct the firm performance Index in Jordanian industrial and service firms.

Variable	Obs.	Mean	Std. Dev	Min	Max
ROA	200	1.30	8.70	-91.90	29
ROE	200	0.99	6.40	-36.70	22.36

Consequently, the maximum value is three. This value was given to companies that meet all of the criteria and then translated (scaled) into a percentage. Finally, all data for each firm were pooled to create the combined datasheet. Due to the nature of each feature that was utilised to generate the index, binary scoring is regarded as adequate for assessing the corporate social responsibility index. Furthermore, Nerantzidis [2] verified that binary scoring is regarded as ideal in a study aimed at presenting compliance scores for businesses.

## Ethics Statement

The Authors declare that the work did not involve the use of human subjects nor animal experiments.

## CRediT authorship contribution statement

**Moawiah Awad Alghizzawi:** Conceptualization, Methodology, Data curation, Software, Writing – original draft. **Mayada A. Youssef:** Supervision, Writing – review & editing, Funding acquisition. **Mohammed Abu Zraiq:** Conceptualization, Methodology, Software, Data curation, Writing – original draft.

## Declaration of Competing Interest

The authors declare that they have no known competing financial interests or personal relationships which have, or could be perceived to have, influenced the work reported in this article.

## Appendix Supplementary materials

Image, application 1
